# The clinical manifestation and neural correlates of cognitive-motor interference in Parkinson’s disease

**DOI:** 10.1038/s41531-026-01464-y

**Published:** 2026-07-08

**Authors:** Jingjing Wu, Jingwen Chen, Cheng Zhou, Xiaojun Guan, Tao Guo, Haoting Wu, Chenqing Wu, Jiaqi Wen, Jianmei Qin, Xiaojie Duanmu, Qianshi Zheng, Weijin Yuan, Zihao Zhu, Baorong Zhang, Xiaojun Xu, Minming Zhang

**Affiliations:** 1 Department of Radiology, The Second Affiliated Hospital, Zhejiang University School of Medicine, Hangzhou, China; 2 Department of Neurology, The Second Affiliated Hospital, Zhejiang University School of Medicine, Hangzhou, China

**Keywords:** Parkinson's disease, Basal ganglia, Cognitive ageing

## Abstract

Motor maintenance in Parkinson’s disease (PD) requires cooperation between cognitive and motor function. This study aimed to explore the manifestation and structural correlates of cognitive influence on PD motor. Forty-four normal controls (NCs), 265 probable isolated rapid eye movement behavior disorders patients (pRBD) and 176 PD patients were evaluated with quantitative motor tests. Subject performance under different task complexities was explored. Some subjects were further examined with magnetic resonance imaging (MRI) and analyzed with cognitive-motor interference (CMI) model concentrated on the subcortical regions. We demonstrated that PD patients had difficulty in performing tasks involving both upper and lower limbs, irrespective of task complexities; and as task became harder, they performed worse. However, during early parkinsonian period, subjects with higher educational attainment were associated with less severe motor dysfunction. Further, our findings revalidated the vital role of substantia nigra played in PD pathophysiology and explored the complex and variable association between subcortical structures and CMI across prodromal and clinical stages of parkinsonism.

## Introduction

Parkinson’s disease (PD) is one of the most common neurodegenerative disorders, which is characterized by complex clinical symptoms, especially motor dysfunction that stands out as the most important characteristic signs of parkinsonism^1^. Its manifestations in the upper and lower limbs lead to limitations in daily activities, such as writing, using devices, and walking, thereby significantly affect personal independence and quality of life^2–5^.

Motor maintenance is a procedure not only involved with motor function, but requires the cooperation of motor and cognition^6^. In daily life, a single motor task is often accompanied by another task, such as talking, thinking, or observation. Additional cognitive demand, such as during dual-task condition, may overload cognitive-motor systems, thereby interfering with the motor task, the cognitive task, or both^5,7^. Former studies exhibited that cognitive load could exacerbate motor disturbance in healthy older people; however, this negative effect is more obvious in patients with PD^4,8,9^. Furthermore, specific tasks aimed at improving cognitive function can improve PD walking performance and hand dexterity^10,11^. It appears that cognition plays an important role in PD motor maintenance. Exploring the neural correlates of cognitive influence on PD motor may deepen our understanding of the neural features of PD and may provide new strategies for clinical treatment.

Notably, patients with isolated rapid eye movement behavior disorders (RBD) are recognized as a specific prodromal state of PD. It was reported that at least 50% of RBD would convert to PD after approximately ten years of follow-up^12^. Classic neuropathological findings support this phenomenon, in which α-synuclein initially deposits in the brainstem and spreads in a caudal-to-rostral pattern during PD progression. This process sequentially involves locus coeruleus (LC) and substantia nigra (SN), explaining why RBD symptoms emerge before motor symptoms^13^. Former studies suggested that the motor disturbance in PD is stemmed from the dopamine depletion and then is mainly involved with the basal ganglia-thalamic-cortex and cerebellum-cortex circuits^14,15^. And functional studies have suggested that the cortex especially the cognitive control regions could compensate for basal ganglia dysfunction on PD motor^16,17^. Often overlooked, other subcortical structures can also exert important influence on PD motor. Impaired cholinergic availability from the basal forebrain (BF) may lead to reduced walking speed in PD^18^, and is associated with PD dementia^19^. The atrophy of BF, amygdala, and hippocampus is indicated as biomarkers of cognitive decline and is correlated with gait dysfunction in PD^20–24^. The cerebellum has also been implicated in cognitive function in PD^25^. Motor maintenance in PD is therefore correlated with multiple subcortical structures, exploring its structural correlates of cognitive-motor interference (CMI) in PD is essential, which may broaden our understanding of PD motor dysfunctions.

**Fig. 1:**
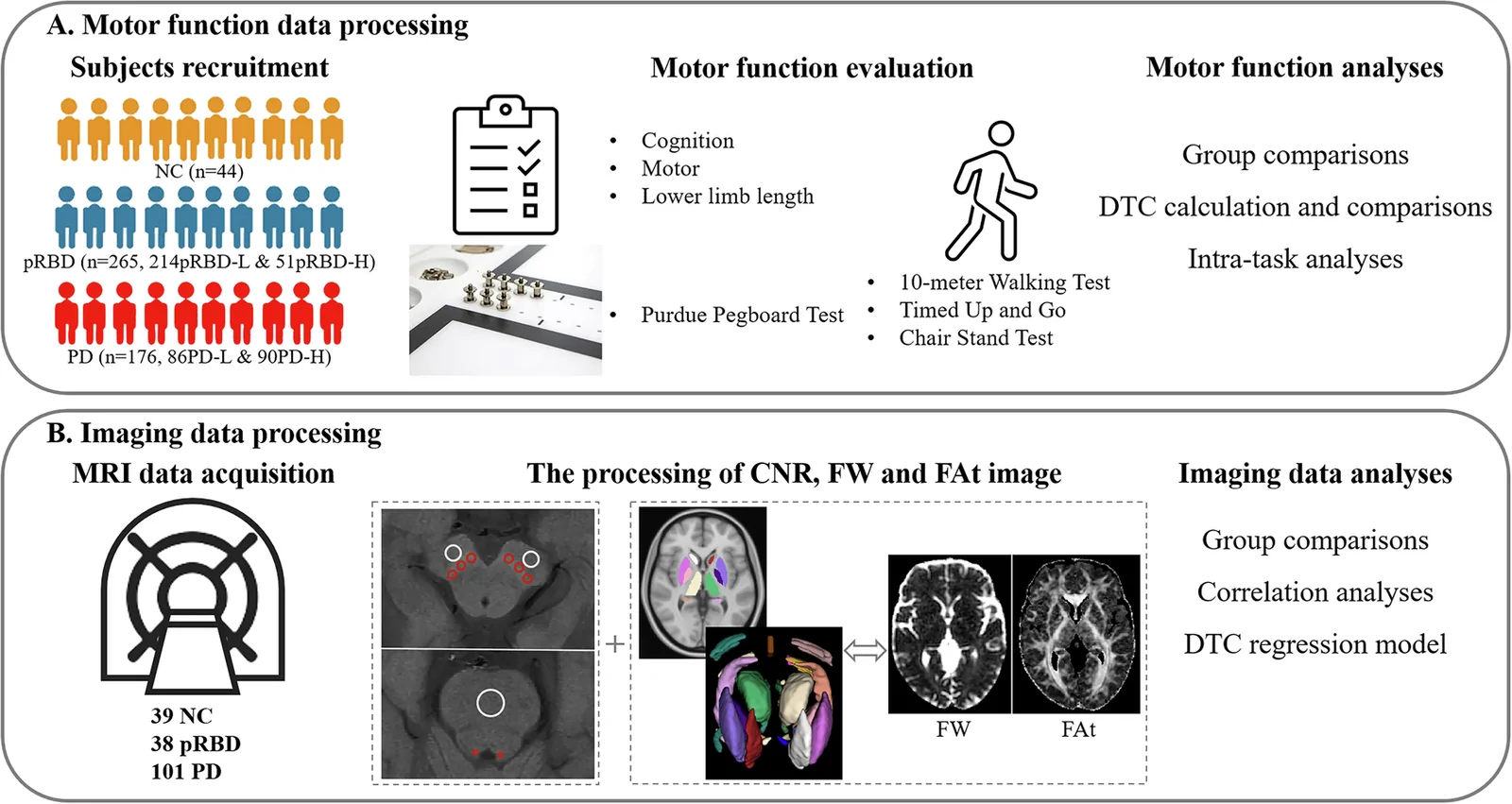
The flowchart of data processing. **A** The motor function data processing procedures, including subject recruitment, motor function evaluation, and motor function analyses. **B** The imaging data processing procedures. There were mainly two parts of imaging processing, including the delineation of LC and SN from NM-MRI, and metrics acquisition from DTI (diagram of a subset of ROIs). LC locus coeruleus, SN substantia nigra, NM-MRI neuromelanin-sensitive magnetic resonance imaging, DTI diffusion tensor imaging, ROIs regions of interest, NC normal controls, pRBD probable isolated rapid eye movement behavior disorders, pRBD-L pRBD with lower education level, pRBD-H pRBD with higher education level, PD Parkinson’s disease, PD-L PD with lower education level, PD-H PD with higher education level, DTC dual-task cost, CNR contrast-to-noise ratio, FW free-water, FAt FW corrected fractional anisotropy.

In this study, diffusion tensor imaging (DTI) was used to detect the microstructural alterations in subcortical structures^26^, and neuromelanin-sensitive magnetic resonance imaging (NM-MRI) was used to reflect pigment cell damage in SN and LC^27^. These MRI measures are considered as sensitive imaging biomarkers for evaluating neurodegeneration in PD patients. We aimed to characterize upper- and lower-limb motor performance in RBD and PD patients across varying task complexities, and employ MRI to identify the subcortical structural alterations, attempting to explore the manifestation and structural correlates of cognitive influence on PD motor (Fig. 1).

## Results

### Demographics of clinical variables

Demographics and clinical variables for all subjects were collected (Supplementary Table S1). No significant difference was found among NC, pRBD, and PD groups for age (*p* = 0.948), while sex (*p* = 0.026) and education (*p* < 0.001) were different. Among the clinical variables, MoCA scores (*p* < 0.001) were reduced in pRBD and PD patients, while RBDQ-HK scores, including total (*p* < 0.001), factor 1 (*p* < 0.001) and factor 2 scores (*p* < 0.001) were higher in PD and pRBD subjects, especially in pRBD subjects, compared with NCs. Notably, education is an important regulatory factor for PD motor function^28,29^, and its level was unbalanced among NC, pRBD and PD subjects of our cohort; therefore, we further separated our pRBD and PD patients to subjects with higher (pRBD-H, PD-H) and lower (pRBD-L, PD-L) education level. A 9-year cut-off was adopted, as it was compliant with China’s 9-year compulsory education system and had been applied in former studies about neurodegenerative diseases^30,31^. Beneath this cut-off, the education level among NC, pRBD-H and PD-H subjects was balanced (*p* = 0.095, Table 1). In total, 51 pRBD-H, 214 pRBD-L, 90 PD-H, and 86 PD-L were categorized. The differential educational attainment of our subjects may be accounted for the role of education played in the self-awareness of physical examination in healthy older Chinese citizens^32^.

**Table 1:** The demographics and motor function data of subjects

	NC	pRBD		PD		*p*			
		pRBD-L	pRBD-H	PD-L	PD-H	NC vs. pRBD-H vs. PD-H	NC vs. pRBD-L vs. PD-L	pRBD-L vs. pRBD-H	PD-L vs. PD-H
Num	44	214	51	86	90				
age	63.25 ± 6.88	63.34 ± 6.93	61.87 ± 7.40	63.51 ± 7.37	63.08 ± 7.51	0.573^d^	0.926^a^	0.133^e^	0.704^f^
sex (M/F)	15/29	95/119	42/9	43/43	57/33	**<0.001**^b^	0.225^b^	**<0.001**^b^	0.074^b^
education	11.48 ± 3.92	3.46 ± 2.81	10.37 ± 1.84	4.42 ± 2.74	11.23 ± 2.33	0.095^a^	**<0.001**^a^	**<0.001**^e^	**<0.001**^e^
Lower limb length (cm)	92.18 ± 5.40	89.61 ± 5.22	92.91 ± 5.24	89.92 ± 5.98	92.27 ± 5.67	0.645^a^	**0.034**^a^	**<0.001**^e^	**0.006**^e^
MoCA	24.80 ± 3.58	15.64 ± 6.00	22.22 ± 4.27	18.16 ± 5.60	23.18 ± 4.39	**0.014**^a^	**<0.001**^a^	**<0.001**^e^	**<0.001**^e^
RBDQ-HK total score	10.02 ± 8.25	33.07 ± 13.75	35.82 ± 15.91	21.12 ± 16.29	17.54 ± 15.05	**<0.001**^a^	**<0.001**^a^	0.307^e^	0.089^e^
RBDQ-HK factor1	7.66 ± 5.39	15.69 ± 6.99	15.12 ± 6.35	10.38 ± 6.29	8.30 ± 6.02	**<0.001**^a^	**<0.001**^a^	0.564^e^	**0.018**^e^
RBDQ-HK factor2	2.36 ± 4.69	17.43 ± 9.28	20.73 ± 12.02	10.74 ± 12.10	9.24 ± 10.62	**<0.001**^a^	**<0.001**^a^	0.093^e^	0.595^e^
Disease duration	–	–	–	4.29 ± 3.69	4.60 ± 4.06	–	**–**	**–**	0.689^e^
H-Y	–	–	–	1–3	1–3	–	**–**	**–**	0.249^b^
UPDRS III	–	–	–	26.83 ± 11.93	24.07 ± 14.01	–	**–**	**–**	**0.035**^e^
PPT right hand	16.04 ± 1.74	13.62 ± 2.37	15.03 ± 2.45	12.03 ± 2.41	11.97 ± 3.13	p1:0.248, **p2:<0.001, p3:<0.001**^c^	**p4:0.001, p5:<0.001, p6:<0.001**^c^	**<0.001**^c^	0.976^c^
PPT left hand	14.72 ± 1.63	12.94 ± 2.24	13.86 ± 2.29	11.08 ± 2.53	11.33 ± 2.88	p1:0.466, **p2:<0.001, p3:<0.001**^c^	p4:0.112, **p5:<0.001, p6:<0.001**^c^	**0.001**^c^	0.347^c^
PPT Both hands	12.37 ± 1.67	10.45 ± 1.99	11.55 ± 1.98	8.57 ± 2.16	9.15 ± 3.78	p1:0.569, **p2:<0.001, p3:<0.001**^c^	p4:0.032**, p5:<0.001, p6:<0.001**^c^	**<0.001**^c^	0.153^c^
PPT total score	42.12 ± 6.11	36.90 ± 6.22	40.17 ± 6.27	31.42 ± 6.53	32.07 ± 8.16	p1:0.682, **p2:<0.001, p3:<0.001**^c^	p4:0.094, **p5:<0.001, p6:<0.001**^c^	**<0.001**^c^	0.289^c^
PPT Assembly	25.20 ± 4.71	18.74 ± 5.05	22.80 ± 6.16	16.64 ± 4.96	18.27 ± 6.82	p1:0.259, **p2:<0.001, p3:<0.001**^c^	**p4:<0.001, p5:<0.001, p6:<0.001**^c^	**<0.001**^c^	**0.033**^c^
CST (s)	9.53 ± 2.46	9.64 ± 2.21	9.20 ± 2.47	13.08 ± 4.02	12.32 ± 4.60	p1:0.601, **p2:<0.001, p3:<0.001**^c^	p4:0.994, **p5:<0.001, p6:<0.001**^c^	0.875^c^	0.246^c^
TUG (s)	7.47 ± 1.43	7.55 ± 1.68	6.84 ± 1.83	10.22 ± 3.50	9.65 ± 3.46	p1:0.211, **p2:<0.001, p3:<0.001**^c^	p4:0.665, **p5:<0.001, p6:<0.001**^c^	0.106^c^	0.165^c^
Mean A (s)	7.80 ± 1.16	7.81 ± 1.33	7.44 ± 1.50	9.54 ± 2.16	9.31 ± 2.84	p1:0.717, **p2:<0.001, p3:<0.001**^c^	p4:0.791, **p5:<0.001, p6:<0.001**^c^	0.759^c^	0.720^c^
Mean B (s)	8.69 ± 1.60	9.48 ± 2.72	8.62 ± 2.15	11.39 ± 3.16	10.77 ± 4.31	p1:0.943, **p2:0.001, p3:<0.001**^c^	p4:0.812, **p5:0.001, p6:<0.001**^c^	0.228^c^	0.703^c^
Mean C (s)	10.61 ± 2.58	10.87 ± 3.46	10.64 ± 4.22	13.22 ± 3.72	12.65 ± 5.79	p1:0.716, p2:0.054, **p3:0.013**^c^	p4:0.481, **p5:0.001, p6:<0.001**^c^	0.677^c^	0.726^c^
DTC B	11.36 ± 12.36	21.41 ± 26.01	16.06 ± 19.01	20.18 ± 24.67	14.53 ± 17.55	0.709^a^	**0.020**^a^ **(p4:0.016**, p5:0.142, p6:1.000)	**0.031**^e^	0.158^e^
DTC C	35.83 ± 26.12	39.40 ± 37.13	41.91 ± 41.93	40.52 ± 36.43	34.68 ± 35.44	0.627^a^	0.965^a^	0.901^e^	0.373^e^

Bold values indicate statistically significant differences. For comparisons of quantitative motor tests among three groups, Bonferroni correction was applied. The post-hoc analysis of DTC B was conducted by Holm–Bonferroni Correction. Effect sizes and 95% confidence intervals for the mean difference between-group comparisons are presented in Supplementary Table S2.

*M* male, *F* female, *MoCA* Montreal Cognitive Assessment, *RBDQ-HK* RBD questionnaire-Hong Kong, *H-Y* Hoehn–Yahr Scale, *UPDRS III* Unified Parkinson’s disease rating scale motor part, *PPT* Purdue Pegboard Test, *CST* Chair Stand Test, *TUG* Timed Up and Go, *Mean A/B/C* the time taken for walking test A/B/C, respectively, *p1* difference between NC and pRBD-H, *p2* difference between NC and PD-H, *p3* difference between pRBD-H and PD-H, *p4* difference between NC and pRBD-L, *p5* difference between NC and PD-L, *p6* difference between pRBD-L and PD-L.

^a^Kruskal–Wallis test.

^b^Chi-Squared Test.

^c^GLM.

^d^ANOVA.

^e^Mann–Whitney Test.

^f^Two-sample *t*-Test.

### Motor function data analyses

For group comparisons, both upper and lower limb performance, including PPT, TUG, CST and walking, were worse in PD patients compared with NCs or pRBD (Supplementary Table S1). This worse tendency also existed for PD-H compared with NCs or pRBD-H, and for PD-L compared with NCs or pRBD-L (Table 1, Supplementary Table S2). The PPT right hand and assembly performance were worse for pRBD than NCs (*p* = 0.001, and *p* = 0.002, respectively) and were also worse for pRBD-L than NCs (*p* = 0.001, and *p* < 0.001, respectively). However, no significant difference of upper or lower limb motor performance was observed between education-matched pRBD-H and NC subjects. For comparisons of groups with different education levels, the pRBD-L group exhibited worse performance than pRBD-H group on all PPT subtests (*p* = 0.001 or *p* < 0.001), with Cohen’s d ranging from 0.57 to 0.94 (Supplementary Table S2). The PD-L group performed worse on PPT Assembly test compared with PD-H (*p* = 0.033), with a Cohen’s d of 0.33. No statistically significant differences for TUG, CST, or walking performance were found between groups with different education levels.

For intra-task comparisons among PPT and walking subtests, we found NC and pRBD-H subjects did not perform differently among PPT subtests (*p* = 0.523, and *p* = 0.923, respectively). In contrast, subjects around total pRBD, PD, pRBD-L, PD-L, and PD-H groups, exhibited different manifestations among PPT tasks with differing complexities (*p* < 0.001, *p* < 0.001, *p* < 0.001, *p* = 0.033, and *p* < 0.001, respectively). Among three walking subtests, we found NC subjects performed differently (*p* < 0.001). For eliminating this worse tendency as shown in normal older people, and to magnify the effects exerted by pathological condition, the individual raw scores of pRBD and PD subjects were transformed to its z’ scores using the means and standard deviations (SDs) of the control group as former studies^33–35^: z’ score = (crude score_pRBD or PD_-mean_NC_) / SD_NC_ (1)_._ Afterwards, we found that pRBD-H subjects did not perform differently among walking subtests (*p* = 0.613). Whereas, subjects around whole pRBD, PD, pRBD-L, PD-L, and PD-H groups performed differently among different walking conditions (*p* < 0.001 for all).

As for DTC parameters, we found that pRBD had greater DTC for walking test B compared with NCs (*p* = 0.045). This tendency was also observed for pRBD-L subjects compared with NCs (*p* = 0.016, *r* = 0.15; the effect size of Kruskal–Wallis test was reported as *r* which was calculated as the absolute value of the test statistic Z divided by the square root of the total sample size, Supplementary Table S2), but not between pRBD-H and NC groups. Furthermore, we found that pRBD-L subjects had greater DTC B than pRBD-H (*p* = 0.031, *r* = 0.13). However, there was no significant difference in DTC B between PD patients and NC or pRBD groups, nor between PD subjects with different education levels. Notably, no significant differences for DTC C were observed regardless of arbitrary group comparisons (Table 1).

### Group comparisons of imaging data and correlation analyses

No significant differences were found for age (*p* = 0.711) or education (*p* = 0.087) among MRI-scanned subjects of NC, pRBD, and PD groups, while sex (*p* = 0.001) showed significant difference (Table 2).

**Table 2:** The demographics and clinical variables of subjects under MRI scanning

	NC	pRBD	PD	*p*
Num	39	38	101	
Age	63.18 ± 6.87	61.85 ± 6.98	62.57 ± 7.49	0.711^a^
Sex (M/F)	14/25	29/9	62/39	**0.001**^**b**^
Education	11.08 ± 3.58	9.83 ± 2.16	10.50 ± 2.35	0.087^a^
Lower limb length (cm)	91.77 ± 5.07	92.36 ± 5.63	92.13 ± 5.63	0.664^a^
MoCA	24.74 ± 3.72	22.16 ± 3.94	23.29 ± 4.21	**0.022**^**a**^
RBDQ-HK total score	10.00 ± 8.27	38.08 ± 15.87	18.04 ± 14.78	**<0.001**^**a**^
RBDQ-HK factor1	7.64 ± 5.54	14.87 ± 5.71	8.61 ± 5.86	**<0.001**^**a**^
RBDQ-HK factor2	2.36 ± 6.68	23.18 ± 12.07	9.43 ± 10.65	**<0.001**^**a**^
Disease duration	–	–	4.63 ± 4.15	–
H-Y	–	–	1–3	–
UPDRS III	–	–	23.41 ± 12.92	–

^a^Kruskal–Wallis test.

^b^Chi-Squared Test.

Bold values indicate statistically significant differences.

**Fig. 2:**
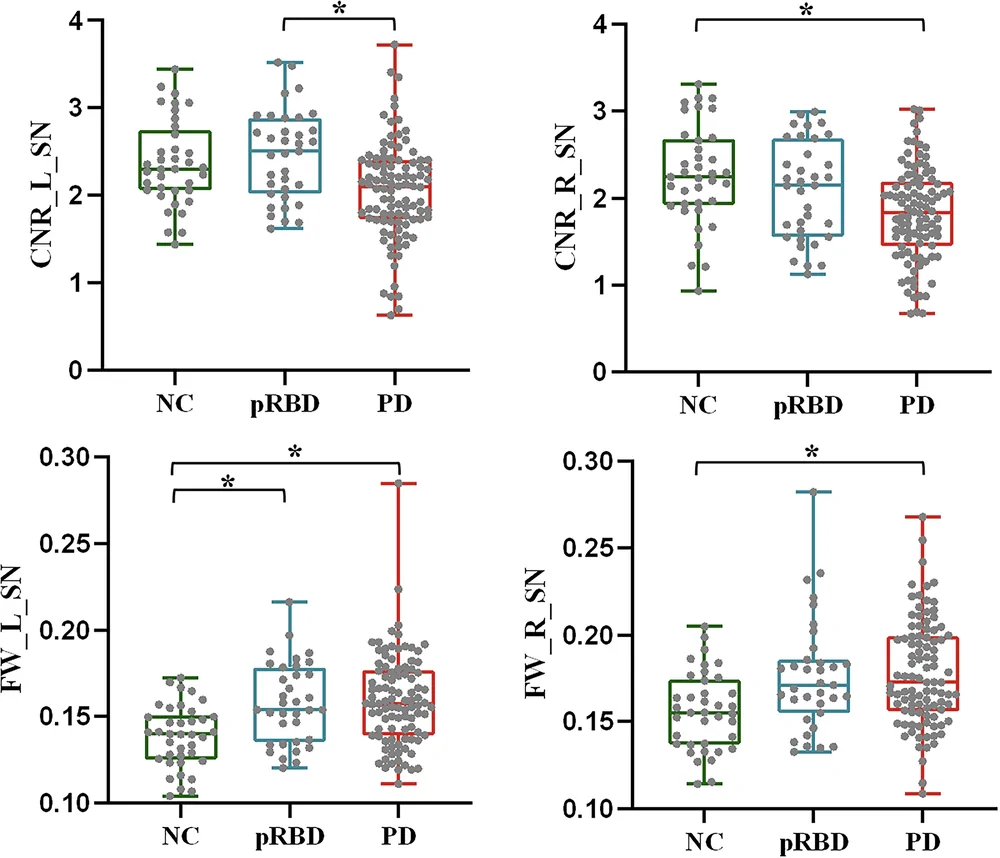
The significant results of imaging data. The significant results of group comparisons. * Denotes the significant results under FDR correction. L left, R right.

For group comparisons of imaging data, we found that the CNR of right SN was lower in PD compared with NCs (*p* = 0.001), and the CNR of left SN was lower in PD compared with pRBD (*p* < 0.001), while no difference was observed between NC and pRBD groups (*p* = 0.345 for left, and *p* = 0.579 for right). The FW of left and right SN was both higher in PD (*p* < 0.001 for both), and the FW of left SN was higher in pRBD (*p* < 0.001) compared with NCs. The FW of bilateral SN was insignificant between pRBD and PD groups (*p* = 0.948 for left, and *p* = 0.818 for right). The significant results are displayed in Fig. 2.

For later correlation analyses, we found that in PD subjects, only correlation between the FW of right SN (*r* = 0.278, *p* = 0.007) and UPDRS III scores could survive correction for multiple correlations (Bonferroni corrected *p* < 0.05/6 ≈ 0.008, as three imaging metrics exhibited between-group differences and two clinical metrics in total). Correlation between the CNR of left SN and MoCA scores (*r* = 0.219, *p* = 0.035), and correlation between the CNR of right SN and UPDRS III scores (*r* = -0.229, *p* = 0.027) for PD subjects were insignificant. In pRBD subjects, only correlation with MoCA was explored. We found that the correlations between CNR of SN with MoCA scores (*p* = 0.169 for left, and *p* = 0.434 for right) or FW of SN with MoCA scores (*p* = 0.405 for left, and *p* = 0.683 for right) were insignificant.

### DTC regression model

At first, DTC model was constructed around each group for DTC B and DTC C, respectively, the significant independent variables of each model were recorded (Supplementary Table S3). For model validation, leave-one-out cross-validation (LOOCV) was applied. However, these results were relatively limited and should be regarded as hypothesis-generating. Nonetheless, the top three most frequently selected variables across LOOCV folds (Supplementary Table S4) were consistent with those identified in the original model (Supplementary Table S3). Detailed procedures and results are provided in the supplementary materials.

**Fig. 3:**
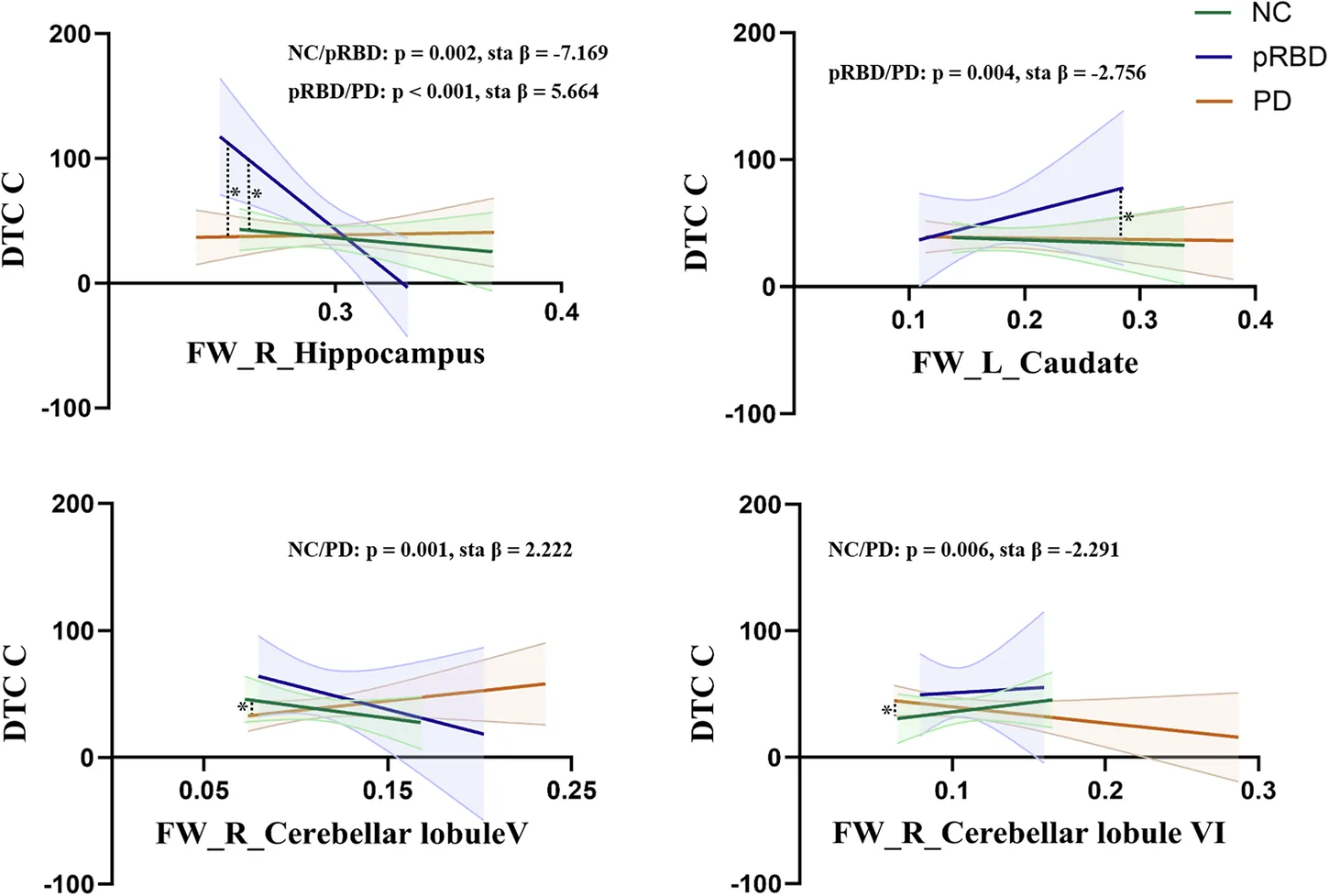
Significant results of the interaction effect of imaging metrics by group on DTC B and DTC C. *Denotes the effect of imaging variable (X) on DTC B or DTC C (Y) was different between these two groups (*p* < 0.017). DTC B, dual-task cost for walking test B; DTC C, dual-task cost for walking test C; sta β, Standardized β.

These independent variables were preliminarily screened for later group comparisons, which employed for exploratorily investigating its modulatory difference between groups. As a result, we found that there were no significant interactions between imaging metrics and group on DTC B. All these results failed to pass Bonferroni corrections. As for DTC C, we found that the effect of the FW of right hippocampus (*p* = 0.002, Standardized β = −7.169) on DTC C was different between NC and pRBD groups. The effect of the FW of right cerebellar lobule V (*p* = 0.001, Standardized β = 2.222) and lobule VI (*p* = 0.006, Standardized β = −2.291) on DTC C was different between NC and PD groups. The effect of the FW of right hippocampus (*p* < 0.001, Standardized β = 5.664), and the FW of left caudate (*p* = 0.004, Standardized β = −2.765) on DTC C was different between pRBD and PD groups. These significant results are presented in Table 3 and Fig. 3.

**Table 3:** Significant results of the interaction effect of imaging metrics by group on DTC B and DTC C

Y	Groups	X	*p*	Standardized β	Adjusted ΔR^2^	Model *p*
DTC C	NC/pRBD	FW_R_Hippocampus	0.002*	−7.169	0.096	0.012
	NC/PD	FW_R_Cerebellar lobule V	0.001*	2.222	0.057	0.001
		FW_R_Cerebellar lobule VI	0.006*	−2.291		
	pRBD/PD	FW_R_Hippocampus	<0.001*	5.664		
		FW_L_Caudate	0.004*	−2.765		

Significant independent variables entered in DTC regression model were collected (X) first, then its interaction term by group (group*X) was introduced into the model. A significant interaction term indicates that the effect of imaging variable (X) on DTC (Y) was different between these two groups.

*Adjusted ΔR²* the change in adjusted R² attributed to the introduction of interaction terms, *BF* basal forebrain.

* and bold values denotes the results under Bonferroni correction with *p* < 0.05/3 ≈ 0.017.

## Discussions

In the present study, we aimed to explore the manifestation and structural correlates of cognitive influence on PD motor from clinical and imaging view. We sought to provide new insights into how cognition influences PD motor, which will be potentially useful for future clinical trials and practice.

Normal subjects can automatically perform movements of relatively complex tasks by integrating multiple brain resources^36^. In contrast, PD patients have more difficulty in performing automatic movements and require more brain-processing resources^4,36^. Therefore, they performed poorly in motor tasks, like the dysfunction presented in both upper and lower limb tasks in our study. However, our whole pRBD or pRBD-L subjects only demonstrated dysfunction in some PPT subtests, rather than in any lower limb tests, including CST, TUG and walking tests, suggesting dysfunction may be more prominent in upper limbs. Elvan et al.^4^ reported that upper limb skills are impaired earlier than lower limb skills, possibly because upper limb skills require higher cognitive demand than lower limb skills, such as balance and walking, which follow a more automatic pattern^37^. These findings indicate the hypothesis that patients in prodromal period may still maintain certain ability to perform motor movements; however, this ability diminishes in clinical stage.

When people attempt to perform complex tasks, the cognitive load becomes heavier, and the performance generally becomes impaired. In this study, we found that increasing cognitive load had a detrimental effect on hand skills and walking performance in PD subjects, which manifested as patients performed worse when task became more complex. Our findings were comparable with former studies, that the impact of dual task on swing times, step length asymmetry, walking speed, stride frequency, and turning is significantly greater for PD than controls^38,39^. Another study demonstrated that the negative effect of dual task on PPT, TUG, and 10MWT is not only greater in PD patients, but is correlated with patients’ quality of life^4^. These findings indicate that PD patients could not balance cognitive and motor performance. Interestingly, the present study found greater DTC of walking test B in pRBD subjects, however, no significant difference was observed for PD patients. Further, neither pRBD nor PD subjects exhibited greater DTC of walking test C compared with NCs. It should be noted that the DTC C was considerable for each group, including controls, which could account for its group insignificance. A previous study exhibited relatively the same results that both older adults and PD patients exhibit significantly worse performance during dual task balancing^40^ and found that the negative DTC on lower limb skills is similar between healthy older subjects and mild-stage PD patients^4^. It seems that when confronting complex tasks, both healthy older subjects and PD patients could not reallocate cognitive resources between tasks, thus, contributes to the deficits of motor automaticity. Furthermore, levodopa can ameliorate simple task straight walking but not complex walking in PD patients^41^, indicating a narrowing of the difference between single and dual tasking performance. Consistent with the findings of Beuter et al.^42^, long term levodopa administration would disturb the observation of walking performance in PD. Overall, these findings demonstrate that the interference between cognition and motor is a widespread phenomenon among all people, especially when people confront with complex conditions^36,39^. Advanced and refined quantitative measurement methods can help identify imperceptible alterations in PD patients. Moreover, evaluating drug-naive individuals in future may provide a more precise reflection of actual disease severity.

In our study, we found that only pRBD-H subjects maintained motor performance regardless of increased task complexities, which was comparable with NC. These subjects were further found to have better performance in all PPT subtests than pRBD with lower education level. These findings indicated that subjects with different education levels may act differently in quantitative motor tests. Multiple studies have emphasized that patients with higher education level are associated with milder parkinsonian motor symptoms^28,29,43^, despite greater reductions in dopamine levels^43^. This could be supported by the finding that more educated pRBD subjects exhibited less DTC of walking test B in this study. It seems that prodromal patients with higher education level are associated with better motor performance and lower interference effect relative to patients with lower education level. However, this association could be limited, as only probable prodromal patients had this manifestation in our study. Previous research has demonstrated that premorbid educational attainment exerts a protective effect against levodopa-induced dyskinesia in PD patients, and this influence diminishes as disease progresses^28^. Another study showed that higher education is associated with better baseline motor function in PD, but not with the rate of motor decline^29^. These evidences strengthen the association of higher educational attainment with less severe motor dysfunction in early parkinsonian period. It should be noted that the floor effect of PD, whereby patients performed poorly in all quantitative motor tests, would also contribute to the limited association between education and PD motor. Education is not an independent factor, but is closely interacted with social, lifestyle, and cognitive conditions. Thus, education may be better to be viewed as a probable surrogate marker of overall status, not a direct proxy for cognitive reserve. In the present study, other factors were not fully controlled for, thus, its underlying interaction effect with confounding factors could not be excluded. Nonetheless, this exploratory association finding may provide new directions for PD strategy in the future.

The genesis and development of PD result from the death of dopaminergic cells within the SN pars compacta that project to the striatum of the basal ganglia, then leading to parkinsonian symptoms. Group comparisons showed that the CNR and FW of SN were both damaged in PD patients. Whereas, among pRBD subjects, only FW of SN was found to be increased than controls, the CNR was relatively intact, indicating that the alteration of FW may be earlier than pigment cell damage during the disease course of PD. Neuromelanin is a complex polymeric pigment that is mainly synthesized in the dopaminergic neurons of the SN and the noradrenergic neurons of the LC^44^. Former studies indicated that the CNR of SN is damaged in PD^45^ and is correlated with disease severity^46^. But the unaltered CNR of LC in pRBD and PD was different from observations in former studies^45,47^. Wang et al.^46^ also found that the CNR of SN is reduced but CNR of LC is unaltered among PD patients without freezing of gait (FOG). Further, some studies suggested that the area under the curve of SN CNR to discriminate PD patients from NC is higher than LC CNR^46,48^. These evidences shed light on the vulnerability of SN in PD and indicate the sensibility of FW for detecting neurodegeneration. Multiple MRI studies suggested that the FW value of SN could be used as a reliable monitoring biomarker for PD^49^. The FW value of SN is found to be correlated with clinical severity^50,51^, can predict longitudinal changes in PD^52^, and is not affected by antiparkinsonian medication^53^, highlighting it as a sensitive and stable disease biomarker. We therefore revalidated the SN to be critical in PD, and its structural alteration was one of the core pathophysiological mechanisms in PD.

Functional studies have given rise to the hypothesis that the competition for brain resources, including cognitive or motor, or the competition for processing mechanisms, can explain worse performance under complicated conditions^36,54^. Although people with PD would require more activity in the motor and cognitive systems than healthy controls to complete complex tasks^17^, motor function cannot be maintained when activation reaches its maximum capacity^16,36^. It was thought that numerous brain regions play an important role in motor maintenance^55,56^; however, the specific structural basis of poorer motor performance in PD remains scarcely known. A former structural study suggested that the subcortical pedunculopontine nucleus connectivity is critical for PD walking^57^. These observations indicate that the regulation of CMI is complicated. In this study, we did a preliminary exploration and found that the structures related to CMI were complex and varied across groups, including hippocampus, caudate, and cerebellar lobules. Only when the task became sufficiently difficult did this regulatory distinction emerge. The atrophy of hippocampus is indicated as the biomarker of cognitive decline in PD, and is correlated with gait dysfunction^21–23^. The caudate nucleus, which is part of the basal ganglia-thalamic-cortex circuit, is important for the motor automaticity in PD^14^. However, it should be noted that the hippocampus and caudate were found to be specifically involved in CMI among pRBD subjects. Former study has shown that, relative to NCs, RBD individuals exhibit reduced functional signal changes in caudate nucleus in response to increased cognitive load, which may account for their dual-task walking deficits^58^. And another study exhibited that PD patients with probable RBD symptoms demonstrate hippocampal atrophy and that is correlated with cognitive deficits, which is not observed in patients without probable RBD symptoms^59^. These findings suggest that hippocampus and caudate may be important structures related with cognitive and gait functions in RBD patients. Apart from the above structures, cerebellar lobules were found to have an important correlates with PD motor. Cerebellum participates in the regulation of automatic posture control^60^, and its activation is increased as task became harder in PD^16,61^. The glucose metabolism in cerebellar lobule V is found to be negatively correlated with motor function, and the metabolism in lobule VI is found to be negatively correlated with cognitive function, with significant differences across cognitive stages^62^. Numerous studies have indicated this compensatory nature of cerebellum in PD. However, emerging evidence suggested that both compensatory and pathological cerebellar processes play a role in PD gait^63^. For instance, one study reported that PD patients with FOG exhibit greater cerebellar gray matter atrophy than patients without FOG^64^. Additionally, PD patients with fall show significantly reduced gray matter volumes in cerebellar lobule V, while smaller volume in lobule VI among non-fall patients is associated with longer reaction times in Trail Making Test parts A^65^. The pathological alterations in cerebellum might be induced by dopaminergic disfunction, resulted from the basal ganglia degeneration or the dopaminergic treatment^63^. Its regulation during parkinsonian period may be curvilinear and a longitudinal study in the future may help reveal this complex association. We can draw at least the conclusion that cerebellar structures are important for PD motor and have a specific correlates with CMI regulation in PD. Specifically, we found that for interference model constructed around each group, SN was only involved in CMI among PD patients, although its effect did not show group difference, the metric of which was further found to be correlated with motor performance in PD. This finding revalidates the important role of SN played in PD and indicates that dopaminergic nigra may be important for CMI regulation. Therefore, it can be tentatively inferred that the motor maintenance during parkinsonian period may involve multiple subcortical structures and the imaging correlates underlying CMI are complex, with its involved structures varied across prodromal and clinical stages of parkinsonism. Our preliminary but worthwhile investigation of CMI in PD highlights that extensive research is warranted in the future to further clarify its underlying pathogenesis.

Some limitations of our study should be noted. First, according to the second edition of the International Classification of Sleep Disorders, PSG is essential for the diagnosis of RBD. However, our study relied on scale assessments, which may increase the possibility of false positive cases when compared to PSG-confirmed RBD. Consequently, the findings related to RBD should be interpreted with appropriate caution. Secondly, this study was a cross-sectional design, which limited its causal interpretation. A longitudinal design would provide more robust evidence regarding the dynamic changes over time. However, data collection for a longitudinal study is time-consuming because of the clinical nature of RBD conversion. And follow-up work has just been initiated for our pRBD cohort. Additionally, the cross-validation results were unsatisfactory and the potential reasons for this were analyzed. First, the small sample size of some groups may lead to the variability in LOOCV iterations; second, some subcortical structures may nonlinearly relate to DTC. It should be noted that the stepwise regression model and LOOCV yielded moderate explained variance, which would impose inherent methodological limitations on results interpretation. Nonetheless, we believe that the present study holds clinical value and can provide novel insights into the manifestations and underlying neural correlates of CMI across PD progression. In the future, we may recruit additional subjects or collaborate with multiple centers to address the sample size bottleneck. More precise model, combined with a longitudinal study would further clarify the dynamic, potentially nonlinear, changes during the parkinsonian periods.

In conclusion, our study demonstrated that PD patients had difficulty in performing tasks involving both upper and lower limbs, irrespective of task complexities; and as task became harder, they performed worse. However, during early parkinsonian period, subjects with higher educational attainment were associated with less severe motor dysfunction. Further, our study revalidated the role of SN played in PD pathophysiology, and indicated its probable role in the regulation of CMI in PD. Not only the dopaminergic nigra, but also cerebellar and hippocampus regions were likely involved in CMI in PD. The underlying structural correlates of CMI were complex and variable across parkinsonian periods. Overall, our findings provided preliminary yet further insights into the clinical manifestations and neural correlates of CMI in PD, thereby advancing our understanding of PD motor dysfunctions.

## Methods

### Subject recruitment

Four hundred and eighty-five subjects were prospectively recruited, including 44 normal controls (NCs), 265 probable RBD (pRBD), and 176 PD patients. All subjects signed the informed consent forms according to the guidelines of the Medical Ethics Committee of the Second Affiliated Hospital of Zhejiang University School of Medicine (Approval No. YAN 2016-082), which was in accordance with the Declaration of Helsinki. pRBD subjects were diagnosed based on RBD questionnaire-Hong Kong (RBDQ-HK)^66^. This questionnaire contains 13 questions, Q1-Q5 and Q13 are assigned to factor 1 regarding dream content, while Q6-Q12 are assigned to factor 2 regarding dream-enacting behavior. The RBDQ-HK factor 2, located at 7/8 points, is validated with polysomnography (PSG) in east China as useful method to screen for RBD^66,67^, with high sensitivity of 90% and specificity of 82%. Thus, in our study, the score ≥8 of RBDQ-HK factor 2 was used to identify pRBD. In addition, PD patients were diagnosed by an experienced neurologist (B.Z.) according to the Movement Disorder Society Clinical Diagnostic Criteria for Parkinson’s disease^68^. Subjects with a history of other psychiatric, neurological or vascular disorders, or brain trauma were excluded.

Age, sex, education, Montreal Cognitive Assessment (MoCA) and RBDQ-HK scores were recorded for all subjects. Disease duration (from the onset of parkinsonian symptoms to clinical evaluation), Hoehn–Yahr Scale (H-Y), and the Unified Parkinson’s disease rating scale motor part (UPDRS III) were specifically recorded for PD patients. For PD patients taking anti-parkinsonian drugs, all examinations were carried out after overnight anti-parkinsonian drugs withdrawal (at least 12 h) to ensure patients were in “OFF” status.

### Motor function evaluation

Motor function was evaluated for all subjects using the Purdue Pegboard Test (PPT)^69,70^, the 10-meter Walking Test (10MWT), the Timed Up and Go (TUG)^71,72^, and the Chair Stand Test (CST)^72^ (Fig. 1A). PPT consisted of four subtests, including right hand, left hand, both hands, and assembly performance. For walking tests, at first, the subjects were required to walk at a comfortable pace over 10-meter distance, named as walking test A. Then, two different cognitive tasks, including reporting odd numbers, and reporting the names of vegetables, were combined with walking, named as walking test B and walking test C. Detailed procedures of all tests please refer to Motor function evaluation in Supplementary Materials. It should be noted that during dual-task trials, subjects were not instructed to prioritize either task; instead, they were asked to walk naturally as usual. This may influence the strategies that subjects adopt during walking, thereby affecting their walking performance. Lower limb length (length from the anterior superior iliac spine to the ground, with shoes off) was recorded for each subject.

The difference in performance between dual-task walking and single-task walking reflects the influence of cognition on walking and the degree of CMI. This can be operationalized as dual-task cost (DTC)^6,73^:

1 $${\rm{DTC}}=\frac{{\rm{Single\; task\; performance}}-{\rm{Dual\; task\; performance}}}{{\rm{Single\; task\; performance}}}\times 100( \% )$$

Therefore, for walking test B and walking test C, DTC was calculated and defined as DTC B and DTC C, respectively.

### MRI data acquisition

Of the recruited subjects, 202 pRBD and 56 PD patients were excluded because of the lack of informed consent or ineligibility for MRI scanning (e.g., limited MRI resources, unwilling to complete long-duration MRI scans, presence of metallic implants, or claustrophobic reactions). In addition, 3 NC, 9 pRBD and 7 PD subjects were eliminated because of poor-quality imaging data. To match the three groups in terms of age and education level, 2 NC, 16 pRBD and 12 PD subjects were further excluded. Finally, 39 NC, 38 pRBD, and 101 age and education matched PD patients were employed for MRI analyses. MRI scanning was conducted with a 3.0 T MRI scanner (GE Discovery 750) equipped with an 8-channel head coil. During scanning, subjects’ heads were stabilized with foam pad, their ears were plugged with earplugs to reduce the noise, and subjects were requested to keep still with their eyes closed. For PD patients, scanning was also executed in OFF-medication status, the same as clinical evaluation.

Structural T1-weighted images were acquired using a fast spoiled gradient recalled sequence: repetition time (TR) = 7.336 ms; echo time (TE) = 3.036 ms; inversion time = 450 ms; flip angle (FA) = 11°; field of view (FOV) = 260 × 260 mm^2^; matrix = 256 × 256; slice thickness = 1.2 mm; 196 continuous sagittal slices. The acquisition plane was parallel to the anterior commissure-posterior commissure (AC-PC) line.

DTI images were scanned using a spin-echo echo-planar imaging sequence with 30 gradient directions (*b*-value = 1000 s/mm^2^): TR = 8000 ms, TE = 80 ms, FA = 90°, FOV = 256 × 256 mm^2^, matrix = 128 × 128, slice thickness = 2 mm, voxel size = 2 × 2 × 2, slice gap = 0 mm, and 67 interleaved axial slices. An additional b = 0 acquisition with reverse phase-encode polarity was acquired for distortion correction. The acquisition plane was parallel to the AC-PC line.

NM-MRI was performed using a T1-weighted fast spin echo sequence: TE = 18.6 ms; TR = 600 ms; FA = 77°; FOV = 220 × 220 mm^2^; matrix = 512 × 512; slice thickness = 3 mm; slice gap = 0 mm; number of slices = 17 (axial). The acquisition plane was orthogonal to the brainstem. Scanning coverage was set from the top of the basal ganglia to the bottom of the medulla oblongata.

### Contrast-to-noise ratio of LC and SN calculations

Two authors (J.W. and C.Z.), who were blinded to subjects’ information, independently performed manual measurements on NM-MRI images. These measurements were processed using ITK-SNAP (https://sourceforge.net/projects/itk-snap/). The contrast-to-noise ratio (CNR) of SN and LC were acquired according to the previous method^74^, detailed procedures please refer to Contrast-to-noise ratio of LC and SN calculations in Supplementary Materials and Fig. 1B. The intra-class correlation coefficient of LC and SN for the intra-rater agreement was 0.908 and 0.803, respectively, which indicated a high consistency.

### DTI data processing

DTI data were processed using the FMRIB Software Library (FSL, http://www.fmrib.ox.ac.uk/fsl) and MRtrix3 (www.mrtrix.org). First, skulls were stripped from the DTI data for each subject. Then, topup and eddy were executed to correct susceptibility-induced distortions, eddy currents, and movements in diffusion data^75^. According to previous study, free-water (FW) was calculated by fitting a bi-tensor model^76^. The signal attenuation of the water molecules caused by extracellular and intracellular water was predicted by this model, respectively. Afterwards, the FW images which reflect the volume fraction of extracellular space water were derived. Further, the component of FW could be eliminated from each voxel to generate FW corrected fractional anisotropy (FAt) map. Then, the FW and FAt of following regions, based mainly on a previous research^77^, were extracted: bilateral SN, BF, amygdala, hippocampus, basal ganglia nuclei (i.e. caudate, putamen, and pallidum), thalamus, cerebellar peduncles (i.e. superior cerebellar peduncle, SCP; middle cerebellar peduncle, MCP), cerebellar lobules V and VI, and cerebellar vermis (detailed procedures please refer to DTI data processing in Supplementary Materials and Supplementary Table S5).

### Statistical analyses of motor function data

Demographics and clinical variables were analyzed in Statistical Product and Service Solutions (SPSS version 23.0). The normal distribution of data was tested by the Kolmogorov–Smirnov test. Differences among groups were analyzed with One-Way ANOVA, Pearson’s chi-square, or nonparametric tests appropriately. *p* < 0.05 was regarded as statistically significant.

In total, there were mainly three aspects of comparisons: 1) Group comparisons of total quantitative motor parameters, which were conducted by General Linear Model (GLM), with age, sex, and education regressed out. Lower limb length was further used as a regressor for 10MWT. And education was not used as a regressor for comparisons between groups with different education levels. For intergroup comparisons among three groups, Bonferroni correction was applied. 2) Comparisons of tasks with different complexities. These intra-task analyses for PPT and walking tests were conducted using ANOVA or Friedman test appropriately, with *p* < 0.05 considered statistically significant. As the scoring scale of PPT subtests was different; therefore, the score of each subtest was first z-transformed. 3) Afterwards, group comparisons of DTC B and DTC C were conducted by nonparametric test. Holm–Bonferroni correction was applied for multi-comparisons.

### Statistical analyses of imaging data

Group comparisons of imaging metrics were conducted by GLM with age, sex, and education regressed out. False discovery rate (FDR) correction was performed for multi-comparisons considering the large number of imaging metrics. Further, the correlations between imaging metrics showing significant group differences and UPDRS III and MoCA were explored with partial correlation analyses. Age, sex, and education were regarded as covariates. Bonferroni correction was adopted for multiple correlations.

### DTC regression model construction

Candidate imaging metrics that could account for DTC were identified using regression model. First, stepwise linear regression model of each group was conducted. DTC B and DTC C were used as dependent variables, respectively. Age, sex, and education were regarded as force-entered covariates in the first block; and all imaging metrics were used as stepwise-entered independent variables in the second block, with enter *p*-value = 0.05 and remove *p*-value = 0.1^78,79^. The model was checked for significant changes after introducing an independent variable (F test). If significant changes occurred, the variable was introduced. *p* < 0.05 was used to evaluate the significance of each constructed model and each introduced independent variable.

Subsequently, an exploration was conducted to examine the intergroup differences in the effect of imaging metrics played on DTC (Y) in SPSS, in other words, the interaction effect of imaging metrics by group on DTC B and DTC C. The significant independent variables entered in the above model were collected, combining group, age, sex, and education, served as multiple X. The group-by-imaging metric interaction term (group*X) was produced and introduced into the model. The results of interaction term with Bonferroni correction (*p* < 0.05/3 ≈ 0.017) were collected. A significant interaction term indicated that the effect of imaging variable on DTC was different between groups.

## Supplementary information

Supplementary materials

## Data Availability

The datasets generated and/or analyzed during the current study are not publicly available due to it was independently collected from our hospital but are available from the corresponding author on reasonable request.
