# Supplementary material for: The clinical manifestation and neural correlates of cognitive-motor interference in Parkinson’s disease
Source: NPJ Parkinsons Dis. 2026 Jul 8;12:267. doi: 10.1038/s41531-026-01464-y (PMC13724707; doi:10.1038/s41531-026-01464-y)
Supplement: Supplementary file 1 [file 41531_2026_1464_MOESM1_ESM.pdf]

Supplementary Materials for

The clinical manifestation and neural correlates of cognitive-motor interference in Parkinson's disease

Jingjing Wu *et al.*

*Corresponding author. Email: zhangminming@zju.edu.cn

Motor function evaluation

The Purdue Pegboard Test (PPT) was administered according to Tiffin and Asher's guidelines.[1] In brief, study subjects were asked to place one cylindrical metal peg into as many of 25 holes in a pegboard as possible in 30s. The test consisted of four subtests, containing using the right, left, and both hands, respectively. And a fourth subtest, named assembly, consisted of assembling pins, collars, and washers within 60s and correctly alternating both hands. Before each subtest, subjects were instructed to put three pins in a hole and perform three assemblies as a part of practice. The total number of pins inserted with the right hand and with the left hand was recorded separately. For both hands simultaneously, the total number of pairs of pins was counted and recorded. Finally, the number of parts assembled was recorded as the assembly score. Each subtest performed three times and the number was averaged afterwards. The averaged number of right, left and both hands was then added up and named as total score. Testing was done by a trained psychologist under constant laboratory conditions. Thus, higher score for PPT indicated better performance.

The 10-meter Walking Test (10MWT) is a commonly used tool for assessing gait performance including the following three subtests: 1) the subjects were required to walk at a comfortable pace over 10-meter distance, named walking test A; 2) the subjects were instructed to report odd numbers continuously while walking, named walking test B; and 3) the subjects were instructed to report the names of vegetables while walking, named walking test C. Each subtest repeated three times, and the time taken to complete each trial was recorded and then averaged.

The Timed Up and Go (TUG) measures the time taken by an individual to stand up from a standard arm chair, walk the distance of 3-meter, turn, walk back to the chair, and sit down again. And the Chair Stand Test (CST) measures the time taken to rise from a chair and sit back down with 5 repetitions. Thus, higher value for 10MWT, TUG, and CST indicated poor performance.

Contrast-to-noise ratio of LC and SN calculations

The substantia nigra (SN) was visible in three contiguous slices of NM-MRI image, in middle slice, which has the greatest area of SN, three regions of interest (ROIs) with a size of 10 mm^2 were placed at isometric lateral, central, and medial SN regions. The adjacent cerebral peduncle (CP) with a size of 30 mm^2 was used as contrast region (**Fig. 1B**).[2] The mean and standard deviation (SD) of the signal intensity (SI) in bilateral SN and CP were calculated. The contrast-to-noise (CNR) of SN was calculated according to $CNR_{SN} = (SI_{SN}–SI_{CP})/SD_{CP}$ (1). The CNR values of lateral, central, and medial ROIs were averaged.

The locus coeruleus (LC) was located at the bilateral areas of the dorsal pons symmetrically, adjacent to the fourth ventricle. Locations with highest SI in three contiguous slices from the level of the inferior colliculi and extending to superior cerebellar peduncles were identified as LC. Circular ROIs were demarcated in bilateral LC and midportions of the pons (PT) at the same slice (as a contrast area).[3,4] The size of ROI was 2 mm^2 for LC and 20 mm^2 for PT (**Fig. 1B**).[5] The mean and SD of SI in bilateral LC and PT were calculated. The CNR of LC was calculated using the following equation: $CNR_{LC} = (SI_{LC}–SI_{PT})/SD_{PT}$ (2). Finally, the CNR value of LC from three slices and two assessments were averaged.

Notably, the ROIs were placed at the predefined anatomical position of SN and LC when the signal was significantly reduced.

DTI data processing

The steps to extract free-water (FW) values were as follows: 1) the native T1-weighted images were linearly and non-linearly aligned into Montreal Neurological Institute (MNI) standard space, and the outputs were visually inspected for errors and bad registration; 2) the b0 images were linearly aligned to native T1-weighted images, and the FW images were projected onto T1-weighted images; 3) the FW images were then linearly and non-linearly aligned into MNI space using the transformation matrix derived from T1-weighted images registration; 4) the derived FW images reflect the volume fraction of extracellular space water, the component of which could be eliminated from each voxel to generate FW corrected fractional anisotropy (FAt) map;

then 5) the FW and FAt of following regions were extracted: bilateral SN, basal forebrain (BF), amygdala, hippocampus, basal ganglia nuclei (i.e., caudate, putamen, and pallidum), thalamus, cerebellar peduncles (i.e. superior cerebellar peduncle, SCP; middle cerebellar peduncle, MCP), cerebellar lobules V and VI, and cerebellar vermis.

For detailed, SN label was obtained from Automated anatomical labelling atlas.[6] The BF (labeled as BF123 and BF4) was defined according to stereotaxic probabilistic maps imbedded in the Statistical Parametric Mapping (SPM, https://www.fil.ion.ucl.ac.uk/spm/) toolbox Anatomy v22c47.[7] Amygdala, hippocampus, basal ganglia nuclei, and thalamus were acquired from Human Brainnetome Atlas (https://atlas.brainnetome.org/). The cerebellar ROIs, including bilateral cerebellar peduncles, cerebellar lobules V and VI, and cerebellar vermis, were hand-drawn on the b-zero image of each subject in Montreal Neurological Institute (MNI) standard space by an experienced rater who was blinded to group membership,[8] and then used to extract the FW and FAt values from the corresponding maps. To alleviate partial volume effects, internal erosion with one voxel were implemented in all ROIs.

Results of dual task cost regression model

At first, dual task cost (DTC) model was constructed around each group for DTC B and DTC C respectively. For NC group, FW of right BF4 ($p = 0.001$), FW of right SCP ($p = 0.009$), and FAt of left cerebellar lobule VI ($p = 0.024$) were correlated with

DTC B (*model* $p = 0.001$, *adjusted* $R^2 = 0.431$, **Table S3**). For pRBD group, FAt of right cerebellar lobule V ($p = 0.014$) was correlated with DTC B (*model* $p = 0.033$, *adjusted* $R^2 = 0.210$). Then, around PD patients, we found that the CNR of right SN ($p = 0.007$), FW of right cerebellar lobule V ($p = 0.001$), and FW of right cerebellar lobule VI ($p = 0.025$) were correlated with DTC B (*model* $p = 0.002$, *adjusted* $R^2 = 0.155$). The same processing was conducted for DTC C. Around NC subjects, FAt of right amygdala ($p = 0.028$) was correlated DTC C (*model* $p = 0.009$, *adjusted* $R^2 = 0.261$). For pRBD, FW of right hippocampus ($p = 0.005$), FAt of right cerebellar lobule V ($p = 0.047$), and FW of left caudate ($p = 0.050$) were correlated DTC C (*model* $p = 0.009$, *adjusted* $R^2 = 0.341$). And the CNR of right SN ($p = 0.001$), FW of right cerebellar lobule V ($p = 0.002$), FW of right cerebellar lobule VI ($p = 0.009$), and FW of right BF123 ($p = 0.023$) were correlated with DTC C (*model* $p < 0.001$, *adjusted* $R^2 = 0.265$) in PD patients.

Validation of DTC regression model

Leave-one-out cross-validation (LOOCV) was used to validate the regression model by using “stepwiselm” in MATLAB. The criterion used to add or remove terms was Bayesian information criterion (BIC). Stepwise linear regression model of each group was conducted. DTC B and DTC C were used as dependent variable, respectively; age, sex, and education as force-entered covariates; and all imaging metrics as stepwise-entered independent variables with enter p-value = 0.05 and remove p-value = 0.1. Each model was validated with LOOCV. Adjusted R^2 and the frequency of each independent variable been selected were recorded. We found that the adjusted R^2 values of each

model are modest (<0.30). Nonetheless, the three most frequently selected variables across LOOCV folds (**Table S4**) were found to be the same as exhibited in the original model. These independent variables were preliminarily screened for later group comparisons, which employed for exploratorily investigating its differed modulatory mechanisms between groups, in other words, exploring the interaction effect of imaging metrics by group on DTC B and DTC C.

References

1 Tiffin, J. & Asher, E. J. The Purdue pegboard; norms and studies of reliability and validity. *J Appl Psychol* **32**, 234-247, doi:10.1037/h0061266 (1948).

2 Ohtsuka, C. *et al.* Changes in substantia nigra and locus coeruleus in patients with early-stage Parkinson's disease using neuromelanin-sensitive MR imaging. *Neurosci Lett* **541**, 93-98, doi:10.1016/j.neulet.2013.02.012 (2013).

3 Li, Y. *et al.* Mild cognitive impairment in de novo Parkinson's disease: A neuromelanin MRI study in locus coeruleus. *Mov Disord* **34**, 884-892, doi:10.1002/mds.27682 (2019).

4 Zhou, C. *et al.* Two distinct trajectories of clinical and neurodegeneration events in Parkinson's disease. *NPJ Parkinsons Dis* **9**, 111, doi:10.1038/s41531-023-00556-3 (2023).

5 Jiang, W. *et al.* Alterations of Interhemispheric Functional Connectivity and Degree Centrality in Cervical Dystonia: A Resting-State fMRI Study. *Neural Plast* **2019**, 7349894, doi:10.1155/2019/7349894 (2019).

6 Rolls, E. T., Huang, C. C., Lin, C. P., Feng, J. & Joliot, M. Automated anatomical labelling atlas 3. *Neuroimage* **206**, 116189, doi:10.1016/j.neuroimage.2019.116189 (2020).

7 Mesulam, M. M., Mufson, E. J., Wainer, B. H. & Levey, A. I. Central cholinergic pathways in the rat: an overview based on an alternative nomenclature (Ch1-Ch6). *Neuroscience* **10**, 1185-1201, doi:10.1016/0306-4522(83)90108-2 (1983).

8 Planetta, P. J. *et al.* Free-water imaging in Parkinson's disease and atypical parkinsonism. *Brain* **139**, 495-508, doi:10.1093/brain/awv361 (2016).

Supplementary Tables

Table S1 The demographics and motor function data at whole group level.

	**NC**	**pRBD**	**PD**	**p**
Num	44	265	176	-
age	63.25±6.88	63.06±7.03	63.29±7.43	0.948[a]
sex (M/F)	15/29	137/128	100/76	**0.026**[b]
education	11.48±3.92	4.79±3.80	7.90±4.25	**<0.001**[a]
Lower limb length (cm)	92.18±5.40	90.25±5.37	91.12±5.93	0.078[a]
MoCA	24.80±3.58	16.92±6.27	20.72±5.60	**<0.001**[a]
RBDQ-HK total score	10.02±8.25	33.60±14.20	19.28±15.72	**<0.001**[a]
RBDQ-HK factor1	7.66±5.39	15.58±6.86	9.31±6.22	**<0.001**[a]
RBDQ-HK factor2	2.36±4.69	18.06±9.93	9.97±11.35	**<0.001**[a]
Disease duration	-	-	4.45±3.88	**-**
H-Y	-	-	1-3	**-**
UPDRS III	-	-	25.41±13.07	**-**
PPT right hand	16.04±1.74	13.89±2.45	12.00±2.79	**p1:0.001, p2:<0.001, p3:<0.001**[c]
PPT left hand	14.72±1.63	13.12±2.27	11.21±2.71	p1:0.103, **p2:<0.001, p3:<0.001**[c]
PPT Both hands	12.37±1.67	10.66±2.04	8.86±3.10	p1:0.134, **p2:<0.001, p3:<0.001**[c]
PPT total score	42.12±6.11	37.52±6.35	31.75±7.39	p1:0.123, **p2:<0.001, p3:<0.001**[c]
PPT Assembly	25.20±4.71	19.52±5.50	17.48±6.02	**p1:0.002, p2:<0.001, p3:<0.001**[c]
CST (s)	9.53±2.46	9.55±2.27	12.69±4.33	p1:0.523, **p2:<0.001, p3:<0.001**[c]
TUG (s)	7.47±1.43	7.41±1.73	9.93±3.48	p1:0.472, **p2:<0.001, p3:<0.001**[c]
Mean A (s)	7.80±1.16	7.74±1.37	9.42±2.52	p1:0.971, **p2:<0.001, p3:<0.001**[c]
Mean B (s)	8.69±1.60	9.31±2.64	11.08±3.79	p1:0.849, **p2:<0.001, p3:<0.001**[c]
Mean C (s)	10.61±2.58	10.83±3.61	12.93±4.89	p1:0.883, **p2:0.005, p3:<0.001**[c]
DTC B	11.36±12.36	20.38±24.87	17.29±21.46	**0.034**[a] (p1:**0.045**, p2:0.405, p3:0.422)
DTC C	35.83±26.12	39.89±38.03	37.53±35.95	0.637[a]

a, Kruskal-Wallis test; b, Chi-Squared Test; c, GLM; p1, difference between NC and pRBD, p2, difference between NC and PD, p3, difference between pRBD and PD. For comparisons of quantitative motor tests among three groups, Bonferroni correction was applied. And the post-hoc analysis of DTC B was conducted by Holm-Bonferroni Correction.

Table S2 Effect sizes and 95% confidence intervals for the mean difference between-group comparisons.

	**p**			
	NC vs. pRBD-H vs. PD-H	NC vs. pRBD-L vs. PD-L	pRBD-L vs. pRBD-H	PD-L vs. PD-H
PPT right hand	p1:0.248, 95%CI (-0.444, 1.707), ES:0.26<br>**p2:<0.001,** 95%CI (2.868, 4.705), ES:1.55<br>**p3:<0.001[c],** 95%CI (2.280, 4.031), ES:1.29	**p4:0.001**, 95%CI (0.703, 2.569), ES:0.81<br>**p5:<0.001**, 95%CI (2.305, 4.204), ES:1.61<br>**p6:<0.001[c]**, 95%CI (1.104, 2.134), ES:0.80	**<0.001[c]**, 95%CI (-2.245, -0.852), ES:0.73	0.976[c], 95%CI (-0.786, 0.763), ES:<0.01
PPT left hand	p1:0.466, 95%CI (-0.616, 1.340), ES:0.16<br>**p2:<0.001,** 95%CI (2.226, 3.896), ES:1.38<br>**p3:<0.001[c]**, 95%CI (1.903, 3.495), ES:1.22	p4:0.112, 95%CI (-0.176, 1.675), ES:0.37<br>**p5:<0.001**, 95%CI (1.713, 3.596), ES:1.33<br>**p6:<0.001[c]**, 95%CI (1.395, 2.415), ES:0.95	**0.001[c]**, 95%CI (-1.788, -0.474), ES:0.57	0.347[c], 95%CI (-1.119, 0.395), ES:0.15
PPT Both hands	p1:0.569, 95%CI (-0.882, 1.600), ES:0.13<br>**p2:<0.001,** 95%CI (1.862, 3.891), ES:1.04<br>**p3:<0.001[c]**, 95%CI (-3.573, -1.552), ES:0.91	p4:0.032, 95%CI (0.075, 1.644), ES:0.51<br>**p5:<0.001**, 95%CI (1.981, 3.578), ES:1.64<br>**p6:<0.001[c]**, 95%CI (1.488, 2.353), ES:1.13	**<0.001[c]**, 95%CI (-1.879, -0.766), ES:0.78	0.153[c], 95%CI (-1.567, 0.247), ES:0.22
PPT total score	p1:0.682, 95%CI (-2.298, 3.504), ES:0.09<br>**p2:<0.001**, 95%CI (6.544, 11.497), ES:1.37<br>**p3:<0.001[c]**, 95%CI (6.057, 10.779), ES:1.28	p4:0.094, 95%CI (-0.367, 4.628), ES:0.39<br>**p5:<0.001**, 95%CI (5.195, 10.279), ES:1.43<br>**p6:<0.001[c]**, 95%CI (4.229, 6.984), ES:1.04	**<0.001[c]**, 95%CI (-5.640, -2.169), ES:0.74	0.289[c], 95%CI (-3.168, 0.948), ES:0.16
PPT Assembly	p1:0.259, 95%CI (-1.040, 3.842), ES:0.25<br>**p2:<0.001**, 95%CI (4.165, 8.332), ES:1.13<br>**p3:<0.001[c]**, 95%CI (2.861, 6.834), ES:0.88	**p4:<0.001**, 95%CI (1.908, 5.812), ES:0.91<br>**p5:<0.001**, 95%CI (4.116, 8.090), ES:1.44<br>**p6:<0.001[c]**, 95%CI (1.166, 3.320), ES:0.53	**<0.001[c]**, 95%CI (-5.847, -2.823), ES:0.94	**0.033[c]**, 95%CI (-3.417, -0.148), ES:0.33
CST (s)	p1:0.601, 95%CI (-1.189, 2.048), ES:0.12<br>**p2:<0.001**, 95%CI (-4.137, -1.338), ES:0.75<br>**p3:<0.001[c]**, 95%CI (-4.480, -1.855), ES:0.86	p4:0.994, 95%CI (-1.262, 1.271), ES:<0.01<br>**p5:<0.001**, 95%CI (-4.819, -2.219), ES:1.29<br>**p6:<0.001[c]**, 95%CI (-4.226, -2.821), ES:1.29	0.875[c], 95%CI (-0.763, 0.651), ES:0.03	0.246[c], 95%CI (-0.553, 2.144), ES:0.18
TUG (s)	p1:0.211, 95%CI (-0.388, 1.743), ES:0.28<br>**p2:<0.001**, 95%CI (-2.872, -1.029), ES:0.81<br>**p3:<0.001[c]**, 95%CI (-3.491, -1.764), ES:1.09	p4:0.665, 95%CI (-1.227, 0.784), ES:0.10<br>**p5:<0.001**, 95%CI (-3.913, -1.850), ES:1.33<br>**p6:<0.001[c]**, 95%CI (-3.218, -2.103), ES:1.23	0.106[c], 95%CI (-0.094, 0.973), ES:0.27	0.165[c], 95%CI (-0.297, 1.729), ES:0.22
Mean A (s)	p1:0.717, 95%CI (-0.732, 1.062), ES:0.08<br>**p2:<0.001**, 95%CI (-2.287, -0.757), ES:0.75<br>**p3:<0.001[c]**, 95%CI (-2.409, -0.965), ES:0.83	p4:0.791, 95%CI (-0.775, 0.592), ES:0.06<br>**p5:<0.001**, 95%CI (-2.527, -1.138), ES:1.24<br>**p6:<0.001[c]**, 95%CI (-2.116, -1.365), ES:1.18	0.759[c], 95%CI (-0.366, 0.501), ES:0.05	0.720[c], 95%CI (-0.803, 0.556), ES:0.06
Mean B (s)	p1:0.943, 95%CI (-1.445, 1.343), ES:0.02<br>**p2:0.001**, 95%CI (-3.272, -0.893), ES:0.66<br>**p3:<0.001[c]**, 95%CI (-3.154, -0.910), ES:0.64	p4:0.812, 95%CI (-1.376, 1.079), ES:0.06<br>**p5:0.001**, 95%CI (-3.359, -0.864), ES:0.79<br>**p6:<0.001[c]**, 95%CI (-2.637, -1.289), ES:0.74	0.228[c], 95%CI (-0.329, 1.372), ES:0.20	0.703[c], 95%CI (-0.877, 1.298), ES:0.06
Mean C (s)	p1:0.716, 95%CI (-1.711, 2.488), ES:0.08<br>p2:0.054, 95%CI (-3.552, 0.032), ES:0.37<br>**p3:0.013[c]**, 95%CI (-3.838, -0.458), ES:0.45	p4:0.481, 95%CI (-2.137, 1.008), ES:0.17<br>**p5:0.001**, 95%CI (-4.404, -1.208), ES:0.82<br>**p6:<0.001[c]**, 95%CI (-3.105, -1.378), ES:0.66	0.677[c], 95%CI (-0.937, 1.440), ES:0.07	0.726[c], 95%CI (-1.211, 1.735), ES:0.05
DTC B	0.709[a]	**0.020[a]** (**p4:0.016**, r:0.15; p5:0.142, r:0.11; p6:1.000, r:0.04)	**0.031[e]** (r:0.13)	0.158[e]
DTC C	0.627[a]	0.965[a]	0.901[e]	0.373[e]

For clinical metrics analyzed with general linear model, effect size was expressed as Cohen's d (indicated as ES). And for nonparametric between-group comparisons (Kruskal-Wallis test), the effect size was reported as r, which calculated as the absolute value of the test statistic Z divided by the square root of the total sample size.

Table S3 The results of DTC regression model.

Domain	Adjusted R^2	Model p	F	Imaging metrics	Standardized β	p
DTC model around only NC subjects						
**DTC B**	0.431	**0.001**	5.419			
				FW of right BF4	0.607	**0.001**
				FW of right SCP	-0.378	**0.009**
				FAt of left cerebellar lobule VI	-0.310	**0.024**
**DTC C**	0.261	**0.009**	4.095			
				FAt of right amygdala	-0.375	**0.028**
DTC model around only pRBD subjects						
**DTC B**	0.210	**0.033**	3.061			
				FAt of right cerebellar lobule V	-0.447	**0.014**
**DTC C**	0.341	**0.009**	3.676			
				FW of right hippocampus	-0.542	**0.005**
				FAt of right cerebellar lobule V	-0.332	**0.047**
				FW of left caudate	0.344	**0.050**
DTC model around only PD subjects						
**DTC B**	0.155	**0.002**	3.840			
				CNR of right SN	-0.270	**0.007**
				FW of right cerebellar lobule V	0.450	**0.001**
				FW of right cerebellar lobule VI	-0.335	**0.025**
**DTC C**	0.265	**<0.001**	5.797			
				CNR of right SN	-0.322	**0.001**
				FW of right cerebellar lobule V	0.417	**0.002**
				FW of right cerebellar lobule VI	-0.373	**0.009**
				FW of right BF123	0.239	**0.023**

Table S4 The three most frequently selected independent variables across LOOCV folds.

Group	DTC B		DTC C	
	Variable	Frequency (%)	Variable	Frequency (%)
NC	FW_BF4_R	100.00	FW_SCP_R	91.67%
	FW_SCP_R	100.00	FWFA_Caudate_L	91.67%
	FWFA_CerebellarlobuleVI_L	94.44	FWFA_Amygdala_R	72.22%
pRBD	FWFA_CerebellarlobuleV_R	96.88	FW_Hippocampus_R	93.75%
	FW_LC_tract_R	68.75	FW_Caudate_L	93.75%
	FWFA_Thalamus_L	68.75	FWFA_CerebellarlobuleV_R	84.38%
PD	SN_R_CNR	100.00	SN_R_CNR	100.00%
	FWFA_Entorhinal_R	93.62	FW_CerebellarlobuleV_R	69.15%
	FWFA_Amygdala_R	88.30	FW_CerebellarlobuleVI_R	69.15%

Frequency, the frequency of being chosen across LOOCV folds.

Table S5 The total image metrics and ROIs.

Metrics	ROIs
**CNR**	LC, right SN, left SN
**FW**	left SN, right SN, left BF123, right BF123, left BF4, right BF4<br>left amygdala, right amygdala, left hippocampus, right hippocampus<br>left caudate, right caudate, left putamen, right putamen<br>left pallidum, right pallidum, left thalamus, right thalamus<br>left cerebellarlobule V, right cerebellarlobule V<br>left cerebellarlobule VI, right cerebellarlobule VI<br>left SCP, right SCP, left MCP, right MCP, vermis
**FAt**	left SN, right SN, left BF123, right BF123, left BF4, right BF4<br>left amygdala, right amygdala, left hippocampus, right hippocampus<br>left caudate, right caudate, left putamen, right putamen<br>left pallidum, right pallidum, left thalamus, right thalamus<br>left cerebellarlobule V, right cerebellarlobule V<br>left cerebellarlobule VI, right cerebellarlobule VI<br>left SCP, right SCP, left MCP, right MCP, vermis
